# Sporadic and Familial Variants in NF1: An Explanation of the Wide Variability in Neurocognitive Phenotype?

**DOI:** 10.3389/fneur.2020.00368

**Published:** 2020-05-05

**Authors:** Maëlle Biotteau, Sébastien Déjean, Sandrine Lelong, Stéphanie Iannuzzi, Nathalie Faure-Marie, Pierre Castelnau, François Rivier, Valérie Lauwers-Cancès, Eloïse Baudou, Yves Chaix

**Affiliations:** ^1^ToNIC, Toulouse NeuroImaging Center, University of Toulouse, Inserm, UPS, Toulouse, France; ^2^Children's Hospital, Toulouse-Purpan University Hospital, Toulouse, France; ^3^Institut de Mathématiques de Toulouse, UMR5219 Université de Toulouse, CNRS UPS, Toulouse, France; ^4^UMR 1253, iBrain, University of Tours, INSERM, Tours, France; ^5^Department of Medicine, University of Tours Francois Rabelais, Tours, France; ^6^Pediatric Neurology, Clocheville Children's Hospital, Tours University Hospital, Tours, France; ^7^Department of Pediatric Neurology and Reference Center for Language Disabilities, CHU Montpellier, PhyMedExp, University of Montpellier, INSERM, CNRS, Montpellier, France; ^8^Epidemiology Department, Toulouse University Hospital, Toulouse, France

**Keywords:** NF1, child, cognitive profile, sporadic, familial, hereditary, SES

## Abstract

**Background:** Cognitive impairment is the most common neurological manifestation in NF1 and occurs in 30–70% of NF1 cases. The onset and severity of each specific cognitive deficit varies greatly from child to child, with no apparent external causes. The wide variability of phenotype is the most complex aspect in terms of management and care. Despite multiple research, the mechanism underlying the high heterogeneity in NF1 has not yet been elucidated. While many studies have focused on the effects of specific and precise genetic mutations on the NF1 phenotype, little has been done on the impact of NF1 transmission (sporadic vs. familial cases). We used a complete neuropsychological evaluation designed to assess five large cognitive areas: general cognitive functions (WISC-IV and EVIP); reading skills (“*L'Alouette*,” ODEDYS-2 and Lobrot French reading tests); phonological process (ODEDYS-2 test); visual perceptual skills (JLO, Thurstone and Corsi block tests) and attention (CPT-II), as well as psychosocial adjustments (CBCL) to explore the impact of NF1 transmission on cognitive disease manifestation in 96 children affected by NF1 [55 sporadic cases (29♀, 26♂); 41 familial cases (24♀, 17♂)].

**Results:** Familial and Sporadic form of NF1 only differ in IQ expression. The families' socioeconomic status (SES) impacts IQ performance but not differently between sporadic and familial variants. However, SES is lower in familial variants than in the sporadic variant of NF1. No other cognitive differences emerge between sporadic and familial NF1.

**Conclusions:** Inheritance in NF1 failed to explain the phenotype variability in its entirety. IQ differences between groups seems in part linked to the environment where the child grows up. Children with NF1, and especially those that have early diagnoses (most often in inherited cases), must obtain careful monitoring from their early childhood, at home to strengthen investment in education and in school to early detect emerging academic problems and to quickly place them into care.

**Trial Registration:** IDRCB, IDRCB2008-A01444-51. Registered 19 January 2009.

## Highlights

- Familial and Sporadic form of NF1 differ in IQ expression
- SES impacts IQ performance but not differently between sporadic and familial variants
- SES is lower in familial variants than in the sporadic variant of NF1
- No other cognitive differences emerge between sporadic and familial NF1
- Inheritance in NF1 failed to explain the phenotype variability in its entirety.

## Background

### Clinical Features of NF1

Neurofibromatosis type 1 (NF1 or also von Recklinghausen's disease), is a tumor predisposition syndrome characterized by the development of typical cutaneous and ophthalmologic manifestations including cafe-au-lait spots, freckling, dermal neurofibromas and Lisch nodules. NF1 patients may also develop endocrine (early-onset puberty, growth retardation), neurological (learning disabilities, epilepsy), ophthalmological (optic glioma), skeletal (bone dysplasia, scoliosis), cosmetic disfigurement or organ compression due to plexiform neurofibromas and vascular complications (high blood pressure) (1–5). NF1 patients are at increased risk of developing various tumors or characteristic malignancy (malignant peripheral nerve sheath tumor or other malignancies such as intracranial astrocytomas, gastrointestinal stromal tumors, pheochromocytomas, juvenilemonocytic leukemia, leukemia, glioma, rhabdomyosarcoma, and breast cancer) (1, 3, 6).

The NF1 phenotype (clinical presentation) is highly variable in expression (7, 8). First, clinical manifestations are progressive and age dependent. They appear gradually during childhood, from cafe-au-lait macules at birth, to skinfold freckles, then lisch nodules and latter neurofibromas (2). Most of the complications persist into adulthood. Second, even if penetrance is complete in children over 8 years old (9), clinical features range from a very mild manifestation to a very severe form of the disease depending of individuals.

Cognitive impairment is the most common neurological manifestation in NF1 and occurs in 30–70% of NF1 cases (10). Studies characterizing the neuropsychological phenotype of NF1 children have highlighted some strong well-established features (11–17). Clinical studies have revealed a left shift in average IQ, ranging from low to normal IQ. Severe intellectual disability (IQ <70) is however unusual, occurring in only about 5% of patients (10, 18–20). Children with NF1 are also at increased risk for difficulties with specific cognitive functions: attention, executive function, reading, expressive and receptive language, language cues interpretation, working memory, visual spatial perception, psychomotor skills (10–14, 21–26). Many school-age NF1 children also experience marked difficulties in learning and academic areas and also presented with learning disorders (reading, mathematics/arithmetic, and written expression). Impaired, poor performance on reading or spelling tasks, deficits, defects in visual-spatial and visual-perceptual skills are therefore common (12). The onset and severity of each specific cognitive deficit varies greatly from child to child, with no apparent external causes. The authors tried to link radiologic specific features in NF1 such as T2 hyperintensities to cognitive impairment (27). In almost 75% of cases, NF1 children present with T2-hyperintensities (4) located mainly in the basal ganglia, thalamus, brainstem and cerebellum, which usually resolve in early adulthood and probably reflect intramyelinic oedema (28). Although the presence and number of T2-hyperintensities do not seem to be related to possible cognitive disorders, some authors find a correlation between IQ scores and their thalamic (29) or cerebellar (30) localization, with an improvement of IQ score with resolving T2-hyperintensities (31). As T2-hyperintensities do not explain the specific cognitive deficits encountered in NF1, authors try to correlate finer structural cerebral changes as higher cerebral volumes or global altered diffusion without further success. Thus, the neuronal substratum of NF1 cognitive phenotype remains unclear.

These cognitive problems do not worsen with age but do not resolve either. They were among the most common manifestation to negatively affect quality of life in NF1 (32–36), leading to significant impacts upon scholastic performance, vocational and professional guidance (37, 38).

### Diagnosis

Despite advances in understanding the genetics of NF1, clinical diagnoses are often made based on physical characteristics [National Institutes of Health Consensus Development Conference Statement –NIH, 1988 (39) for formal diagnostic criteria for NF1, later reaffirmed in 1997], including cutaneous, ophthalmologic, and orthopedic features (1, 40, 41) see Box 1.

Box 1 **National Institutes of Health (NIH) diagnostic criteria for neurofibromatosis type 1 (NF1) (39).**

The clinical diagnosis is based on the presence of two or more major disease features out of the following:

- Six or more café-au-lait macules >5 mm in greatest diameter in pre-pubescent individuals, and >15 mm in post-pubescent individuals
- Two or more neurofibromas of any type or one plexiform neurofibroma
- Freckling in the axillary or inguinal regions
- Optic glioma
- Two or more iris hamartoma (Lisch nodules)
- Distinctive bony lesion such as sphenoid dysplasia, or thinning of the long bone cortex with or without pseudoarthrosis
- A first-degree relative (parent, sibling, or offspring) with NF1 based on the above criteria

### Genetic

NF1 is one of the most common childhood neurogenetic disorders worldwide, affecting approximately 1 in every 2,500 to 3,500 individuals (42, 43). It is caused by mutations in the NF1 gene, a classic tumor suppressor gene (44) on chromosome 17 (17q11.2) which encodes neurofibromin that is largely expressed in the nervous system (45–47). The most of NF1 gene lesions inhibit the expression of intact neurofibromin. Neurofibromin belongs to a family of proteins that act as negative regulators of the ras oncogene and serves as a tumor suppressor (43, 48, 49). Disruption of neurofibromin explains why NF1 patients are at risk for developing tumors.

NF1 is an autosomal dominantly inherited disease, which means that when one of the parents has the disease, there is a one in two chance of transmitting NF1 to an offspring (1). However, although NF1 is an autosomal dominant condition, about 50 percent of NF1 cases are due to new mutations (42, 50) resulting from a *de novo* mutation. The NF1 gene exhibits a very high new mutation rate [between 3–5 × 10^−5^ and 1.4–2.6 × 10^−4^, (51, 52)], among the highest observed in humans, 10 times higher than is typical for human disease gene loci (53). The NF1 gene is one of the largest human genes, with about 350 kilobases of genomics DNA, containing 61 exons to encode 2,839 amino acids and producing an 11–12.5 kilobase messenger RNA containing an open reading frame of 8,454 nucleotides (45, 54, 55). However, neither the size nor the complexity of the NF1 gene are sufficient to account for this unusually high new mutation rate (56).

Sporadic cases (consequence of new mutations) occur in the absence of a NF1 family history. It may be difficult to distinguish clinically mosaic NF1 individuals from NF1 individuals with an inherited NF1. It has been noted that more than one sporadic case can be observed in several families, each with a distinct NF1 mutation (57, 58). There are also some rare cases of parental mosaicism for an NF1 mutation, comprising germline mutation (59–61). In this situation, the parents do not carry the disease but one of them carries the genetic anomaly in some of their reproductive cells. Such individuals (considering as having a mosaic NF1) have a weaker but unquantifiable risk of passing on generalized NF1 to an offspring. Parental mosaicism is also considered as a sporadic case (62, 63). It should be noted that the frequency of NF1 mosaicism in the population may be underestimated since some mosaic individuals may have no clinical evidence of NF1 (63).

Given that the development of congenital syndromes and cancer predisposition are associated with advanced maternal or paternal age (64), research has been done on NF1 (65–67) but has lead to conflicting reports and lesser association between age and *de novo* mutations. As in several other autosomal dominant disorders, paternal age has been mentioned to explain sporadic cases. However, although the average age of fathers of children with sporadic NF1 was more often higher than fathers in the general population (65, 66), effect of age to *de novo* mutation is small or non-existent (68). In addition, several studies have suggested that 90% of spontaneous mutations in NF1 originate in the paternal genome (46, 69). Kaplan et al. (63) found that mutated allele (R1968X, recurrent variant present in 1 to 2% of individuals with NF1) is of maternal origin.

### Care Management

The wide variability of phenotype both between individuals with NF1 and within NF1 families is the most complex aspect in terms of management and care. Clinical features and presentation of NF1 are extremely variable (7, 8) and involve many of the body systems. The complications of NF1 differ depending on the individual concerned (presence vs. absence of each possible symptom) and variation in their expressions (minimal, mild, moderate, or severe) is considerable and highly heterogenic. In addition, clinical presentation is unpredictable even within the same family (1, 52), including the age of disease onset and the severity of clinical symptoms (70, 71). For example, Upadhyaya et al. (58) report the genetic analysis of a unique family with NF1, in which the three affected members had a different heritable and pathological mutations in their NF1 genes and exhibited different clinical evidence of NF1. In familial form, the severity of manifestations in a child cannot be expected by a parent's clinical course (or other family members) and the risk of having a seriously ill child is 1 in 12 (1). Members of the same family and even more, identical twins with NF1, often exhibit variable syndrome expression. Thus, twin studies, which have been a respected tool for studying genetic disorders, were not conclusive in the NF1 literature reports. Numerous studies have presented monozygotic twins, both affected by NF1 but differing in their phenotypes (62, 72). Kaplan et al. (63) also presented monozygotic twin discordant for autosomal disorder NF1 (unaffected twin that show no clinical manifestations) who is therefore considered as a mosaic even if the distribution of the mutant allele among different cells and tissues seems insufficient to induce clinical manifestations of NF1. Detjen et al. (62) did not detect any mitochondrial DNA differences between individuals of the same twin pair, highlighting that mitochondrial DNA polymorphisms [an obvious candidate for an extrachromosomal phenotype modifier (73)] do not seem to contribute to the phenotypic variability in NF1.

Copy number variants research (CNVs) also failed to understand phenotypic variability (74). In their study, the authors analyze CNVs in 11 pairs of monozygotic twins with several phenotypic discordances and concordances to identify genetic factors potentially affecting disease manifestation but found no differences in CNVs that could justify discordant NF1 characteristics (74).

A recent study (68) found that there is no significant effect of parental age on the incidence of NF1 or the coexistence of different NF1 symptoms, or on their level. The authors also did not find any relationship between sex and clinical symptoms, although previous study have showed that sex can be a key influence for neural dysfunction in NF1 (75).

With the exception of deleting the entire NF1 gene which is associated with a very severe form of disease (76), clinical manifestations are irrespective of the causative genetic alterations (77): most studies thus showed no relationship between the particular NF1 mutation type (whether they are missense/non-sense, point mutations, splicing, micro- or gross-deletions, micro- or gross-insertions, duplications, etc.) and the expression of clinical manifestations in NF1 individuals. Cognitive deficits encountered in NF1 are no exception to this rule and such uncertainty and lack of knowledge on why complications of NF1 differ depending on the individuals concerned is exactly the same for cognitive impairments (78).

This very high variability of the NF1 phenotype, including for individuals carrying the same NFl gene mutation, suggests that other factors (other modifying genes, epigenetic influences, second hit somatic mutations in the NF1 gene, hormonal milieu, but also environmental factors or chance) might be involved in the clinical expression of the NF1 phenotype and might be the reason behind this phenomenon (71, 79, 80). Little is however known about such relative contributions.

The management of NF1 children is therefore mainly based on follow-up, in order to detect possible complications such as behavioral or cognitive deficit as soon as possible since early care significantly improves skills and abilities. This uncertainty is a source of stress and anxiety for families and establishing prognostic factors could help professionals responsible for the follow-up of these patients.

### Aims and Objectives

Despite multiple and serious research, the mechanism underlying the wide variability in NF1 has not yet been elucidated. Prospective identification and screening of such individuals is currently not possible. There is currently no way to measure, predict or know which NF1 patients will develop one or several symptoms but not others (and why), or which NF1 patients will develop complications or not. However, identifying factors that modify the NF1 phenotype may greatly help to improve patient counseling.

Brain development is based on the ongoing interaction of innate biological determinants and environmental determinants that will modulate the organization, structuring and functioning of the brain. In our study, we therefore ask if the mode of transmission (and consequently the environmental determinants) can influence the cognitive phenotype in NF1 patients.

Indeed, while many studies have focused on the effects of high specific and high precise genetic mutations on the NF1 phenotype, little has been done on the impact of NF1 transmission (sporadic vs. familial cases). This question of transmission establishes a solid framework to study environmental factors, the social level in which the child evolves, the detection of disease and diagnosis (which may be earlier in family forms), the difficulties of informing parents of the clinical status of their child and the consequences of such a disclosure [given that pessimism is less common in familial than in sporadic NF1 (36)], etc.

The issue of influence between sporadic or familial onset on cognitive profile of affected NF1 individuals was not extensively discussed. Learning disabilities, which are very frequent in NF1, is however one of the chief factors in the deterioration of quality of life in NF1 patients (10, 36), both in children and adults since they lead to poor school academic performances, preclude individuals to higher education and graduate school, lower the education level and restrict individuals' choices and their professional future. They also cause great damage to self-image, the development of assertiveness and independence.

In this study, we used a large set of cognitive performance abilities (attention, reading, intellectual, and visual-spatial skills), as well as psychosocial adjustments to explore the impact of NF1 transmission (sporadic vs. familial cases) on cognitive disease manifestation in 96 children affected by NF1 [55 sporadic cases (29♀, 26♂); 41 familial cases (24♀, 17♂)].

## Methods

### Participants

The participants included 55 children with sporadic NF1 expression and 41 children with the familial NF1 variant, all aged between 8 and 12 years old. Patients with a family history of neurofibromatosis type 1 or a first-degree relative with one criteria of neurofibromatosis type 1, according to the NIH criteria, were classified as having the familial variant. Patients without any familial history of neurofibromatosis type 1 were classified as having the sporadic variant. Seventy-five of them had been previously included in a published study on the cognitive profile of NF1 (81). NF1 subjects were recruited from the existing NF1 patient population at six French national NF1 referral centers (Children's Hospitals of Lyon, Montpellier, Nantes, Paris, Toulouse, and Tours). Inclusion criteria were (1) age between 8 and 12 years and (2) a confirmed clinical diagnosis of NF1 according to the NIH criteria (39). MRI examination was not required for inclusion but if it had been done, symptomatic optic glioma was considered to be an exclusion criterion. Children with a known major medical, neurological or psychiatric disorder that could potentially affect cognition (epilepsy, brain tumor, hydrocephalus, head injury, autism, or intellectual disability with an IQ below 70) or with uncorrectable hearing or visual impairment were excluded.

All parents and children gave their informed oral and written consent, after the nature and objectives of the study were thoroughly explained. Approval to conduct this study was granted by the French Ministry of Health's Hospital Programme for Clinical Research (PHRC 2008, Toulouse University Hospital, no. 08 113 01), Occitanie Regional Council (APRTC no. 09004813), and the local Ethics Committee (CPP Southwest, France) in accordance with the Declaration of Helsinki convention.

### Procedure

Participation in the study was offered to parents by a pediatric neurologist through a clinic for follow-up. A leaflet describing the characteristics of the study, a recruitment letter, a consent form and a demographic/health screening questionnaire were directly given or sent to them later.

All the children were received for two half-day sessions. They underwent a medical examination to exclude ADHD and other neurological and psychiatric diseases, and to confirm the NF1 diagnosis. Then, all the children underwent the same complete five-part individual, neuropsychological evaluation conducted by certified clinical neuropsychologists, using a comprehensive and large protocol designed to assess five large cognitive areas: general cognitive functions; reading skills; phonological process; visual perceptual skills and attention. Each area was composed of several tests which were given in a specific and the same order as part of a neuropsychological battery. However, the order of administration of the five large cognitive areas was randomly changed between subjects to minimize the order bias of the neuropsychological assessment results (81).

### Measures

This study fits in with a larger project [methodology described in Chaix et al. (81). Each participant was assessed with a comprehensive battery of standardized psychometric tests including (i) all subtests of the Wechsler Intelligence Scale for Children—Fourth Edition [WISC-IV (82)]; and the French version of the “Peabody Picture Vocabulary Test-Revised” [EVIP (83)] for the cognitive assessment; (ii) the “*Alouette*” French reading test [revised version (84)], the ODEDYS-2 test (85) and the ORLEC battery (86) for the reading and phonological skills assessment; (iii) the Judgment of Line Orientation test (87), the Thurstone test and the Corsi blocks for visual perceptual assessment; and (iv) the Conners Continuous Performance Test-Second Edition (88) and the parent form Child Behavior CheckList (CBCL) questionnaire (89) for the attention and psychosocial skills assessment. All the assessments were conducted in French. French norms were used to calculate scores for all children wherever available.

The issue of the socio-cultural characteristics (occupation and educational level of parents) has also been raised.

**Cognitive abilities** were assessed with the French-language version of the WISC-IV (82) designed for children between the ages of 6 to 16. In this psychological assessment, four primary Index make up the Full Scale IQ score (Verbal Comprehension Index -VCI, Perceptual Reasoning Index -PRI, Working Memory Index -WMI, and Processing Speed Index -PSI Scores; themselves calculated from several subtests). The core 10 subtests include Comprehension, Similarities, and Vocabulary subtests for the VCI; Block design, Picture concepts and Matrix reasoning for the PRI; Digit span and Letter-number sequencing for the WMI Coding and Symbol search for the PSI. Raw scores were converted to age-scaled scores (standard scores for subtests M = 10, SD = 3; standard scores for index and full scale IQ: M = 100, SD = 15). All subtests and indexes have demonstrated good reliability and validity and are considered good measures of general intelligence.

**Reading disorders** were evaluated with the “*L'Alouette*” French reading test (84), the most widely used reading test in French-speaking countries. The “*L'Alouette*” test is currently used as the “gold standard” test by health care professionals (especially speech therapists) and researchers to screen for reading level (good or poor readers, dyslexia) among children and adolescents. **Reading text assessment** was completed by word recognition procedures, measured by the “Word Reading” subtest from the ODEDYS-2 test (85). **Phonological processing** (phonological memory and phonemic awareness) was measured with three tests from the ODEDYS-2 battery of tests (85) (1) pseudoword repetition task (phonological short-term memory), (2) phonemic deletion task (phonemic awareness), and (3) blending task or acronyms task (phonemic awareness). **Reading comprehension efficiency** for both sentences and text was assessed through a standardized reading comprehension test, the ORLEC battery (86). The first subtest (L1) consists of text to be read aloud. The second subtest (L3) is silent reading comprehension test with a forced-choice sentence completion test.

**Receptive lexical skills** were determined by the French version of the “Peabody Picture Vocabulary Test-Revised” (EVIP) (83). EVIP is intended to provide a quick estimate of the receptive vocabulary ability of children from the ages of 2.6 to 18 years old.

**Sustained attention and impulsivity capacities** were measured by the Conners Continuous Performance Test-Second Edition (88). CPT-II is a computer-administered neuropsychological task used to evaluate the attentional functioning (sustained attention and impulsivity) of individuals aged at least 6 years old. It is commonly used in research and by clinical means for discriminating inattentive, hyperactive, and impulsive behavior difficulties in children via target vs. non-target stimuli designed to have minimal language and memory demands. The CPT-II leads to 12 outcome measures but we only analyzed the four main scores: Omissions (number of non-responses to target), commissions (number of responses to non-target stimuli), hit reaction time (measure of response speed consistency), and perseverations (measure of response inhibition).

**Psychosocial adjustments** were assessed with the parent form of the Child Behavior CheckList (CBCL) questionnaire (89). The CBCL is a parent-report measure of behavioral and emotional problems for children aged 6 to 18 years used in both clinical and research practice. It lists internalizing and externalizing symptoms of 113 child behaviors.

**Visual perceptual abilities** were assessed with the Judgment of Line Orientation test (JLO) (87) and the Thurstone test. The JLO test measures a person's ability to match the angle and orientation of lines in space using a task that consists of matching two angled lines that appear at the top of a page, to the angles of two lines among a standard fan-shaped array (semicircle) of 11 lines (separated 18 degrees from each other) appearing at the bottom of the page. The Thurstone's Identical Form Test measures certain capacities of visual and spatial perception. For this test, the visual-spatial component is predominant. **Visual-spatial memory** was assessed by determining forward and backward spans with the Corsi blocks-tapping test.

**Socioeconomic status of the parents (SES):** Both parents of the participants were asked about their profession and level of education. We coded the professions into eight categories according to the classification of professions and socio-professional categories established by the National Institute of Statistics and Economic Studies (*Institut national de la statistique et des sciences économiques*, INSEE) in 1982. This statistical classification, that allows professions to be classified, exists at an aggregate level of eight positions (1. Farmers, farm operator; 2. Artisans, traders and entrepreneurs; 3. Employees and higher intellectual professions; 4. Intermediate professions; 5. Employees; 6. Workers 7. Pensioner; 8. Other individuals without activity professional) but is also developed in 24 and 42 positions (with correspondence between the three nomenclatures). This nomenclature allows the grouping of individuals into homogeneous social categories according to their professional activity, on the basis of three main criteria: the hierarchical position within the profession performed (completed by the level of diploma required to practice this profession), the status (employee or self-employed), and the nature of the activity (agricultural or non-agricultural).

To this nomenclature, we added a classification of the level of education in 5 gradients: (1) Without diploma or “*Brevet des colleges”*; (2) CAP or BEP; (3) General, technological or vocational baccalaureate (around 18 years); (4) Diploma 2 years after baccalaureate; (5) Graduate and postgraduate diplomas (Bachelor's degree, Master's degree).

### Statistical Analysis

As a first screening step, statistical tests were conducted for every numerical variable to compare the two sub-groups: sporadic and familial. Both Student and Wilcoxon-Mann-Whitney rank-sum tests were run as a way to ensure the relevance of the results. In order to study the effect of socioeconomic status on the previous results, 5-factor ANOVAs were conducted for each numerical variable. These analyses included the NF1 form as well as the level of education and the profession of the two parents. To make it easier to interpret the results, no interaction factors were included in the models. As the mother's level of education appeared as the SES variable with the highest effect, further investigation focused on this factor jointly with the NF1 form. Two-factor ANOVA with interaction were then conducted to assess the potential cross effect of these two factors. Every analysis was performed using the R software (version 3.5.2, released 2018-12-20).

## Results

### General Characteristic of the Population

The 96 children included in the main analysis were part of this exploratory analysis. Demographic (age, sex) and socioeconomic status variables (level of education and profession of the two parents) are presented in Table 1. The population is roughly balanced between the sporadic NF1 variant (55) and the familial variant (41). This proportion allowed statistical comparison between the two groups to be considered. The two groups were homogeneous with no differences in terms of age or gender. Factors that could influence cognitive ability (socioeconomic status of the father and mother: parental educational level and profession) were also analyzed and were similar across groups concerning father data, but significantly different regarding the mother.

**Table 1:** Demographic and Social Characteristics of the Sporadic and Familial Groups.

	**Sporadic transmission**	**Familial transmission**	***P*-value**
	***N* = 55**	***N* = 41**	
**Demographic characteristics**			
Age in years [Mean (SD)]	9.8 (1.4)	10.2 (1.3)	0.2431
Gender [Boys/Girls]	26/29	17/24	
**Social characteristics**			
**Educational level of father (*****N*****/%)**			**0.2081**
Without diploma	9 (16.4%)	4 (9.8%)	
CAP or BEP	11 (20%)	5 (12.2%)	
Baccalaureate	14 (25.5%)	19 (46.3%)	
Two years after baccalaureate	9 (16.4%)	4 (9.8%)	
Graduate and postgraduate diplomas	7 (12.7%)	8 (19.5%)	
*Missing*	5 (9.1%)	1 (2.4%)	
**Educational level of mother (*****N*****/%)**			0.0212^*^
Without diploma	9 (16.4%)	14 (34.1%)	
CAP or BEP	10 (18.2%)	2 (4.9%)	
Baccalaureate	13 (23.6%)	12 (29.3%)	
Two years after baccalaureate	15 (27.3%)	4 (9.8%)	
Graduate and postgraduate diplomas	6 (10.9%)	8 (19.5%)	
*Missing*	2 (3.6%)	1 (2.4%)	
**Profession of father**			**0.7501**
Farmers, farm operator	2 (3.6%)	3 (7.3%)	
Artisans, traders and entrepreneurs	4 (7.3%)	4 (9.8%)	
Employees and higher intellectual professions	14 (25.5%)	6 (14.6%)	
Employees	10 (18.2%)	10 (24.4%)	
Worker	10 (18.2%)	10 (24.4%)	
Intermediate professions	8 (14.5%)	4 (9.8%)	
Without activity	3 (5.5%)	3 (7.3%)	
*Missing*	4 (7.3%)	1 (2.4%)	
**Profession of mother**			0.0298^*^
Employees and higher intellectual professions	15 (27.3%)	5 (12.2%)	
Employees	21 (38.2%)	18 (43.9%)	
Worker	1 (1.8%)	4 (9.8%)	
Intermediate professions	10 (18.2%)	2 (4.9%)	
Without activity	8 (14.5%)	11 (26.8%)	
*Missing*	0 (0%)	1 (2.4%)	

* *p < 0.001*.

### Clinical and Neuropsychological Results, Differences Between Sporadic and Familial Variants

Numerical variables (*n* = 49) are presented in Table 2. For each variable, mean and standard deviation are displayed as well as the *p*-values of the Student and the Wilcoxon-Mann-Whitney tests. The variables are ordered depending on the test or cognitive processes evaluated.

**Table 2:** Clinical Characteristics of the Sporadic and Familial Groups.

	**Sporadic**		**Familial**			
	**Mean**	***SD***	**Mean**	***SD***	***P*-value (Student)**	***P*-value (Wilcoxon)**
**Efficiency (WISC-IV)**						
FSIQ	93.1	13	85	11.5	0.0016^*^	0.0015^*^
PRI	92.1	12	85.2	10.5	0.0034^*^	0.0048^*^
Block design	8.3	2.8	6.7	2.8	0.0076^*^	0.0038^*^
Picture concepts	9.3	2.4	9	2	0.5594	0.7389
Matrix reasoning	9	2.4	7.4	2.7	0.0039^*^	0.0051^*^
VCI	100.1	13.5	94.5	13.5	0.0464^*^	0.0159^*^
Vocabulary	9.9	2.4	8.9	2.8	0.0595	0.0289
Similarities	10.5	3.2	9.3	2.9	0.0747	0.0261
Comprehension	9.7	2.6	9	2.3	0.2221	0.1448
WMI	90.2	14.1	82.1	14.2	0.0073^*^	0.0085^*^
Letter-number sequencing	8.9	3	7.1	2.6	0.0033^*^	0.0038^*^
Digit span	7.9	2.7	6.9	2.6	0.0898	0.0851
PSI	94.9	11.7	91.9	13.5	0.246	0.1909
Coding	10.6	12.5	8.8	2.9	0.2976	0.5576
Symbol search	9.1	2.6	8.3	3.1	0.1869	0.2025
**Reading speed (Alouette)**						
CTL (SD)	−0.5	1.1	−0.7	1.1	0.3656	0.36
CM (SD)	−1.6	2.4	−1.9	3	0.5554	0.6566
**Reading strategies (ODEDYS)**						
Irregular words reading	−0.3	1.2	−0.4	1.1	0.444	0.3891
Pseudowords reading	−0.9	1.2	−1.1	1.3	0.5068	0.6349
Regular words reading	−0.6	1.5	−0.8	1.9	0.6078	0.8027
Pseudowords reading (duration)	−0.6	1.4	−0.7	1.7	0.6987	0.9117
Irregular words reading (duration)	−0.2	1.4	−0.6	1.7	0.3086	0.4958
Regular words reading (duration)	−0.5	1.6	−0.8	1.7	0.4255	0.5228
**Phonological process (ODEDYS)**						
Phoneme blending	−0.7	1.1	−1	1.2	0.2475	0.1964
Pseudoword repetition	−1.4	2	−1.7	2.2	0.4488	0.4971
Phoneme suppression	−0.3	0.9	−0.2	1	0.7705	0.5626
**Reading comprehension (Lobrot)**						
Sentence comprehension (quartile score)	2.3	1	2	1.1	0.1648	0.1177
Text comprehension (quartile score)	2.4	1	2.1	1.1	0.262	0.1891
**Lexical level (EVIP)**						
Score (SD)	114.3	13.6	110.4	16	0.2204	0.1606
**Attention level (CPT-II)**						
Commission	54	29.1	58.5	25.3	0.4235	0.5895
Omission	61.8	26.1	61.3	25.7	0.9231	0.8949
Perseveration	54	23.2	56	27.5	0.713	0.8275
Hit reaction time	65.3	30.4	63.7	26.2	0.7812	0.5473
Hit reaction time standard error	63	30.6	67	25.2	0.4887	0.675
**Psychosocial (CBCL)**						
Totals problems	−3.4	1.3	−3.4	1.6	0.8675	0.9289
Internalizing	−1.4	1.7	−1.3	1.7	0.8166	0.8705
Externalizing	0	1	−0.3	1.2	0.2656	0.2121
Aggressive behaviors	0	1	−0.3	1.1	0.2232	0.1328
Somatic complaints	−1.1	1.4	−1.4	1.8	0.2724	0.5586
Attention problems	−2.1	1.7	−1.7	1.6	0.2892	0.2908
Throughout problems	−0.8	2	−0.2	1	0.0533	0.319
Social withdrawal	−1	1.7	−0.7	1.5	0.4654	0.3882
Delinquent behaviors	−0.2	1.1	−0.4	1.7	0.5103	0.8937
Social problems	−1.4	1.7	−1.2	1.9	0.6092	0.4508
Anxiety/Depression	−1.2	1.6	−1.1	1.6	0.6417	0.5471
**Visuoperceptual abilities**						
JLO score	−1	1.2	−1.4	1.2	0.0964	0.0955
Thurstone score	0	0.9	−0.2	0.9	0.2048	0.2492
Forward span (Corsi)	0.1	1.3	0	1.4	0.6743	0.8572
Backward span (Corsi)	0.5	1.2	0.5	0.9	0.7652	0.9844

* *p < 0.001. FSIQ, Full Scale IQ; VCI, Verbal Comprehension Index; PRI, Perceptual Reasoning Index; WMI, Working Memory Index; PSI, Processing Speed Index*.

The differences between sporadic and familial variants were investigated in three ways.

First, a screening consisted of performing two sample statistical tests (Table 2) which highlights, on the one hand, the consistency of the results between the two tests, and on the other hand, some variables (ordered below depending on the increasing *p*-values of the Student tests) with significant differences between the sporadic and familial variants: FSIQ, Letter-Number Sequencing, Perceptual Reasoning Index, Matrix Reasoning, Working Memory Index, Block Design, and Verbal Comprehension Index obtained an uncorrected *p*-value lower than 5%. As we are in a multiple testing context, adjusted *p*-values with Bonferroni correction were also calculated. This correction resulted in *p*-values systematically higher than 5%. This means that our results have to be considered with moderation and not as highly significant results. But, the fact remains that the lowest *p*-values are associated with the numerical variables showing differences between the two variants.

Secondly, the effect of SES variables together with the NF1 variant was studied in 5-factor ANOVAs. The results are presented in Table 3 for the IQ variables with differences between both groups (with a *p*-value for the NF1 variant factor lower than 5% in Table 2). It appeared that the numerical variables with the lowest *p*-values for the NF1 variant remained nearly the same as in the first step (FSIQ, PRI, Matrix Reasoning, etc.). In addition, more interestingly, the level of education of the mother was the SES variable with the lowest *p*-value. Let's note also that the Bonferroni correction applied on these *p*-values would give 0.0147 (0.0003^*^49) for the *p*-value of the NF1 variant related to FSIQ. Thus, the effect of the NF1 variant on the FSIQ is significant when SES is taken into account.

**Table 3:** *p*-values for individuals effects (NF1 group, level of education mother and father, profession mother and father) from a 5-factor ANOVA performed on each significant numerical variable.

	**Transmission**	**Educational level of mother**	**Educational level of father**	**Profession of mother**	**Profession of father**
FSIQ	0.0003^*^	0.0135^*^	0.1643	0.7697	0.5235
PRI	0.0016^*^	0.0261^*^	0.2156	0.4637	0.9465
WMI	0.0044^*^	0.711	0.3492	0.9438	0.3373
VCI	0.0056^*^	0.0778^*^	0.6654	0.9303	0.6537
Matrix reasoning	0.0028^*^	0.6458	0.8606	0.4742	0.5788
Block design	0.0056^*^	0.0802^*^	0.0854	0.1498	0.6718
Letter-number sequencing	0.0035^*^	0.5546	0.4163	0.7115	0.3456

* *p < 0.001*.

Thirdly, as the level of education of the mother seemed to be the most important SES variable, we focused on the cross-effects of this factor with the NF1 variant. To address this problem, we ran 2-factor ANOVAs with interactions. The results, presented in Table 4, show that the interaction effect is never significant. This means that the effects of NF1 variant and the level of education of the mother occur independently: the effect of one factor does not depend on the level of the other factor.

**Table 4:** *p*-values for individual effects (NF1 group, level of education mother) and interaction from a 2-factor ANOVA including interaction performed on each IQ variable.

	**Transmission**	**Educational level of mother**	**Interaction**
FSIQ	0.0021^*^	0.015^*^	0.6528
PRI	0.0045^*^	0.0227^*^	0.3516
Block design	0.0125^*^	0.065^*^	0.326
Picture concepts	0.5473	0.046^*^	0.1148
Matrix reasoning	0.0059^*^	0.6793	0.9964
WMI	0.0133^*^	0.6012	0.2884
Letter-number sequencing	0.0073^*^	0.8355	0.3677
Digit span	0.138	0.7085	0.3205
VCI	0.0298^*^	0.1469	0.2034
Comprehension	0.166	0.4325	0.0636
Similarities	0.0403^*^	0.0051^*^	0.2901
Vocabulary	0.0557	0.5358	0.715
PSI	0.2936	0.0283^*^	0.5703
Symbol search	0.2027	0.1724	0.2258
Coding	0.3785	0.4323	0.2128

* *p < 0.001*.

## Discussion

In the current study, we wanted to elucidate if NF1 transmission has an impact on the wide variability on cognitive phenotype in NF1 children. The only difference from a broad battery of neuropsychological tests -including psychometric, reading (text and word), phonological, visual-spatial, reading comprehension (sentences and text), receptive language, attention and psychosocial assessments- was in the IQ scores.

### IQ Differences Between Sporadic and Familial Form of NF1

We detected a highly significant difference between sporadic and familial NF1 cases in all index scores -except Processing Speed Index (PSI)-, Full Scale IQ (FSIQ) and three subtests: Matrix Reasoning, Block Design, and Letter-Number Sequencing.

For the FSIQ, PSI, and WMI, children affected by the familial variant of NF1 were lower than average (standard scores around 80 to 85 for indexes and between 6 and 7 for subtests) while children affected by the sporadic variant of NF1 were average (90 to 95 for indexes and around 9 for subtests) suggesting that patients with sporadic NF1 adapt better to the disease than familial cases.

Our results were in accordance to those of Coutinho et al. (90) that have found better scores in FSIQ, VCI and WMI (the details of the subtests has not been carried out) in children with sporadic NF1 than children with familial NF1. However, our results differ from those of Lehtonen et al. (91), Hyman et al. (10), and Ferner et al. (20) who found similar IQ scores between sporadic and familial variants. Several reasons can be put forward to explain such discrepancies in the results. First, these three studies were designed to compare cognitive profiles between NF1 patients and controls and not between the sporadic and familial variants of NF1. Thus, the authors did not specify if both groups were taken from comparable populations (if number, percentage, age, sex, number of borderline IQ is comparable between groups, if the main confounders were identified and taken into account in the design and analyses to minimize the risk of bias, etc.). Secondly, differences can be due to the sample age. In our study, children are between 8 and 12 years old, while in Hyman et al. (10) and Lehtonen et al. (91), children were older (8 to 16.75 years and 6 to 16 years, respectively) and in Ferner et al. (20), the 103 patients with NF1 (51 sporadic NF1 cases and 52 familial NF1 cases) are between 6 and 75 years (mean age 27.6 y/o; SD 18.2). Genetic influences—that explain significant parts of the observed variation in cognitive functioning, both for children and adults (92, 93)—tend to increase in significance with age, while environmental influences decrease in significance across development (94–96). Brant et al. (94) especially show that the environmental factors that have an influence on variance in intelligence are very minor from age 12 onwards. There is a great similarity of the pattern of contributing factors from between ages 12 and 16, suggesting that the etiology of individual changes in intelligence development is extremely constant by early adolescence. Another and final explanation to such a discrepancy is that, in these three previous studies, mental retardation is not excluded and 6.2% of children with NF1 in Hyman et al. (10), 6% in Lehtonen et al. (91) and 8% in Ferner et al. (20) have an FSIQ <70. The inclusion of extreme cognitive profiles is probably a bias (controlled in our study) that leads to different results.

Over the last two decades, there have been a number of studies, summarized in the systematic review of Lehtonen et al. (12), that have studied the general intellectual functioning of children with NF1. The majority of studies have shown that, although children with NF1 have IQs in the normal range, their IQ is often lower (around 90s) than their peers or than their unaffected siblings (10, 19, 20, 97). However, some studies failed to prove differences in IQ between children with NF1 and norms (29, 98–100). In addition, Lehtonen et al. (12) pointed out a disagreement to the IQ profile of children with NF1: while some studies demonstrated that children with NF1 scored less on all subtests of the WISC, some others detected some significant differences in only some subtests (Block design or Digit span, for example.) between children with NF1 and their siblings. Difference in proportion of transmission (proportion of sporadic vs. familial NF1 in the final sample) variant could perhaps explain these contradictory results.

We found a difference between sporadic and familial NF1 children regarding Block Design (8.3 vs. 6.7, respectively), Matrix Reasoning (9 vs. 7.4) and Letter-Number Sequencing (8.9 vs. 7.1). Block designed^1^ and Matrix reasoning^2^ (moderately correlate each other; *r* = 0.55) are known to be a good measure of general and fluid intelligence abilities. They measure non-verbal reasoning, visual processing and abstract, visual perception and organization, visual-spatial ability (and visual-constructional ability for Block Design). Letter-Number Sequencing^3^ measures attention span, short-term auditory memory processing, sequential processing and mental manipulation (101–104). Those three subtests may be influenced by concentration and attention.

Differences between sporadic and familial groups of NF1 children in those three subtests are very interesting. All three are considered to be the hallmark phenotypic characteristics of patients with NF1: children with NF1 are known to have serious difficulties in visual-spatial abilities, memory and attention (11, 12, 23, 25, 81, 91, 105).

Altogether, it is therefore legitimate to ask whether IQ difference -largely previously proved between NF1 children and peers or unaffected siblings- persist if the modality of transmission is taken into account. Are differences maintained between children affected by a sporadic variant of NF1 and peers and siblings? Do children with the familial variant of NF1 constitute a “bias” or an explanation to the wide variability in cognitive profile of NF1? More research is needed to detail this specific topic. The mode of transmission of NF1 also seems essential to be taken into account in future studies about the cognitive profile of NF1 subjects.

### What Is the Role of Socioeconomic Status (SES) for Such IQ Differences in NF1?

Today, the concept that the cognitive performance of an individual depends approximately equally on his/her genetic heritage and his/her environment is a consensus. Recent genome-wide meta-analyses and research studies have identified genomic loci and genes linked to variation in intelligence (106–111). However, it is also known that the socio-economic background of the child places constraints on their IQ (95, 112). First, indices of the families' SES (education, occupation and income of parents) have been proved to moderate the heritability of their children's intelligence (113–115). The heritability of IQ is higher for children who are raised in high SES environments (115). The results of Turkheimer et al. (114) especially demonstrate that the proportion of IQ variance due to environment and genes change non-linearly with SES: in disadvantaged families, the contribution of genes is close to zero and the environment (SES) explains 60% of the IQ variance, whereas is it the reverse in wealthy families. Secondly, in the general literature, the SES environment has been shown to account for variance in cognitive functioning in childhood in many studies. The effects of the environment on IQ, especially the link between the socio-economic level of parents (socioeconomic status and parental education) and the cognitive performance of children is therefore well-established (96, 115). Of course, the level of education of the child's family environment is involved (especially that of the mother): parents from high SES environments indeed offer more occasions for activities and learning experiences to boost and encourage children's intellectual development (115). But differences in intellectual outcomes could also be attributable to the family income, nutrition, sleep, stress, availability of parents, maternal, and paternal involvement, etc. -that have a direct impact on the child's cognitive development and that is directly connected to the child's environment. For example, concerning income, Noble et al. (116) followed a cohort of 1,099 individuals aged 3 to 20 years. Authors highlighted that income relates most strongly to brain structure (especially in regions supporting language, reading, executive functions and spatial skills) among the most disadvantaged children: small differences in income were associated with large differences in brain surface area in these children, whereas in higher income families, the same differences in income involved smaller differences in surface area). Thirdly, some recent studies tend to highlight the link between IQ and epigenetic mechanisms [temporary (or not) genetic changes supported by environment]. For example, in times of high stress, physiological changes in the organism can modify genes. These modifications can impact a set of features that can have knock-on effects affecting the child development. Kaminski et al. (117) have especially found a relationship between the epigenetic modifications of one specific gene and IQ, indicating experiences have an impact on the genetic mechanisms involved in complex processes such as intelligence. Authors thus show that individual differences in IQ are linked to differences in brain activity and epigenetic changes, which are both under environmental influences.

Altogether, studies have found a strong relationship between IQ and SES in the general population, suggesting our experiences/environment not only affect our quality of life, the wiring of our brain, but the very way our cognitive function evolves.

In the NF1 children population, Hyman, Lehtonen and Ferner's studies have examined predictors of the lowering of general cognitive ability and have only found an association with socioeconomic status. SES has also been found to correlate with general intelligence in Lorenzo et al. (118, 119).

In our study, we have found a strong link between (1) sporadic/familial form, (2) IQ and (3) SES family background, especially the mother's education level.

Firstly, children with familial NF1 had a significantly lower SES than children with sporadic NF1, which is consistent with other NF1 studies (90, 91, 118). Lorenzo et al. (118) especially found in a population of 43 children with NF1 (25 sporadic cases and 18 familial cases) that 68% had mothers who completed a university or postgraduate degree in sporadic cases group compared to 28% in the familial cases group. Coutinho et al. (90) similarly found lower SES in children with the familial transmission than in children with the sporadic transmission (41% vs. 19%). Such distribution does not appear to be an unexpected outcome: the sporadic vs. familial NF1 variant has an impact on the social level in which the child evolves (91, 118). NF1 frequently leads to learning disabilities, poor school academic performances (23), lower education level (less likely to graduate from school, less likely to complete tertiary education), and restrict individuals choice and their professional future (individuals with NF1 are and thus fall into lower socio-economic groups) (120).

Secondly, and as previously shown in the general population (115, 121) and in the NF1 population (10, 90, 118), we found that SES was, in turn, associated with IQ achievement. NF1 children from greater SES backgrounds (here children affected by the sporadic variant) had greater cognition scores than those raised from lower SES backgrounds (here children affected by the inherited variant). Our results are in accordance with Lorenzo et al. (118) and Hyman et al. (10). However, both studies have addressed the issue of the relationship between NF1 in its entirety, IQ and SES (10, 118, 119), without addressing the specific question of the relationship between NF1 variants, IQ and SES. Our results are also in line with Coutinho et al. (90), who found that children with the familial transmission had a lower FSIQ and tended to have a lower SES compared to those with sporadic NF1. However, the authors did not discuss this association (Cause and effect? Consequence? etc.). Our findings therefore increase those of these four previous studies in the comprehension of this trend, highlighting that the disparity recognized between the sporadic and familial variants is likely due to the impact that the NF1 transmission modality has on the SES environment of the family.

In addition, we also demonstrated that there is no significant interaction between group (transmission forms: sporadic vs. familial) and the relationship between the mother's education level and the IQ of the children. In other words, the mother's education level has an impact on the IQ of the NF1 child, irrespective of the transmission mode (sporadic or familial). Having a low SES has a snowball effect on other variables -as cognitive variables- but effect is irrespective of inherited variant. However, as familial NF1 leads more frequently to a low SES, familial NF1 children are most often affected.

### Toward a More Complex and Multi-Factorial Approach to Explaining Specific Cognitive Phenotypes in NF1

Another important finding is the absence of differences for tests exploring the usually affected cognitive domains in NF1 (language, visual-spatial domain, executive functions, attention) between the two forms of NF1. We indeed used 10 tests leading to 49 measures, completed by four SES measures. We only found a single test and only seven measures out of 49 where there is a difference between the sporadic and familial NF1 variants. The majority of cognitive functions are therefore not different between the two groups. Consequently, we can argue that transmission (sporadic vs. familial) alone failed to explain the wide variability in phenotype NF1 expression.

Our results were consistent with those of Coutinho et al. (90). Although authors found that children with sporadic NF1 performed better than those with familial NF1 in a large battery of neuropsychological tests (Reading Comprehension tasks, Rey Complex Figure Copy, Spatial Memory, JLO, Imitation of Hand Positions), differences were canceled when FSIQ and SES were taken into account (except for JLO). Our results were also consistent with those of Lehtonen et al. (91) and Erdogan-Bakar et al. (122) where the heritability status of NF1 did not lead to any differences in the performance of the children with NF1 (sporadic vs. familial NF1 groups) on any of the measures (visual-spatial, working memory, spatial memory, executive function, attention, etc.). Note, however, that these two studies were not designed to observe this effect (this is here an ancillary result), so the groups were not controlled and adjusted in terms of number, age, sex, IQ, SES, etc. Our results therefore reinforce, confirm and extend these previous ones with equivalent groups (no bias), and a study especially designed to reply to this question.

The causes of NF1 cognitive phenotype and its variability have been explored with genetic, brain imaging or histological studies (27) but none have successfully explained them until now [for e.g., (10, 18, 21, 27)]. Snippets of explanation are sometimes pointed (UBOs, visual-spatial abilities, etc.) but findings are inconsistent across studies. The IQ variability could be explained by the transmission (sporadic vs. familial) and SES status, while another variability typology (motor impairment, social deficit, executive function impairment, etc.) could be explained by another cause. It is therefore possible that the wide variability in NF1 can be explained by a multitude of causes and not just one, which would partly explain why studies fail to explain phenotype variability in NF1 when they address this question from just one perspective.

Overall, cause-and-effect relationships to explain phenotype variability in NF1 are not always easy to establish and more global approaches are probably needed. Multi-causality is also a possible explanation that should be investigated: either as interrelated causes that interact in a particular order to produce the effect; or as the interaction of multiple risk factors, including environmental, economic, lifestyle and genetic predisposition factors.

## Conclusion

Altogether, we therefore highlighted (1) that there is an IQ difference between children affected by the sporadic variant compared to the familial variant, (2) that such difference is linked to SES status of the child family, (3) that there is no difference between groups on the impact of SES on IQ, (4) but that there is significantly lower SES in familial NF1 families than in sporadic NF1 families, and (5) that IQ differences between groups seems consequently in part linked to the environment where the child grows up.

The question of NF1 transmission institutes a robust framework to study the impact of environmental determinants and their repercussions on health, care, disease development and prognostics. Inequalities in health reflect the inequalities that can generally be seen within a society. The findings from our study have clinical implications with regard to the management of NF1: children with NF1, and especially those that have early diagnoses (most often in inherited cases), must obtain careful monitoring from their early childhood, at home to strengthen investment in education and in school to early detect emerging academic problems and to quickly place them into care. Our findings also have implications in research that leads to taking care of the effects of inherited and sporadic cases of NF1 in the evaluation of cognitive and behavioral assessments, considering this variable with great interest in developmental studies since it is largely determined by the environment in which the child grows up.

On the other hand, our results imply that the inherited variant of NF1 (familial vs. sporadic) does not explain specific deficits in NF1 (reading, visual-spatial, attention, psychosocial functions). We would strongly encourage research to advance further in the understanding of phenotypic variability in NF1, since this is a major hindrance to the prognosis, monitoring, and care of such patients. However, we believe that researching a unique and common cause to the set of variabilities is not the right solution. This variability exerts on many components (presence or not of physical characteristics, cognitive functions impairments, level of the symptoms, which one and which intensity, presence or not of UBOs, their location, their numbers, etc.) and maybe there is one cause behind each type of component or clusters of components. Appropriately identifying the responsibilities behind each variability has a real and significant interest for health care in NF1.

## Limitation

Further research on larger NF1 populations is needed, and shall include genetic data recovery to allow genotype-phenotype analyses and their correlation to the neurobehavioral phenotype.

## Data Availability Statement

The datasets generated and/or analyzed during the current study are not publicly available due to the nature of the data (interventional research protocol involving the human person) but are available from the corresponding author on reasonable request.

## Ethics Statement

This study is registered with ClinicalTrials.gov number NCT02397967. The study was approved by the local ethics committee (CPP Sud-Ouest Outre-Mer 1) and conducted in accordance with the Declaration of Helsinki. Written informed consent to participate in this study was provided by the participants' legal guardian/next of kin.

## Author Contributions

YC is the principal investigator of the study, conceived the idea for the study, and was a major contributor in writing the protocol. YC, MB, and EB analyzed and interpreted the data, conceived the first working plan based on results, and wrote the manuscript. SD carried out the statistical analysis, wrote the statistical sections of the manuscript, and reviewed the final manuscript. SL was involved in study coordination and quality monitoring. NF-M was involved in study coordination and carried out the neuropsychological tests. SI carried out the neuropsychological tests. PC and FR included patients. VL-C contributed to the writing of the protocol and reviewed the final manuscript. All the authors read and approved the final manuscript as submitted and agreed to be accountable for all aspects of the work.

## Conflict of Interest

The authors declare that the research was conducted in the absence of any commercial or financial relationships that could be construed as a potential conflict of interest.

## Footnotes

^1^BD (core Perceptual Reasoning subtest) require children to put together red-and-white specially designed blocks in a pattern according to a displayed model. The subtest is timed.

^2^MR (core Perceptual Reasoning subtest) require children to complete a matrix or serial reasoning problem by selecting a missing picture from five response choices. The subtest is untimed.

^3^LNS (core Working Memory Subtest) require children to repeat in a predetermined order to the examiner a series of numbers and letters that they just heard. The subtest is untimed.
